# Peroneus brevis rupture associated with a hypertrophic peroneal tubercle: a case report and literature review

**DOI:** 10.1186/1757-1146-6-S1-P14

**Published:** 2013-05-31

**Authors:** Dean Samaras, Andrew Kingsford

**Affiliations:** 1 Kingsford Podiatry Group, Melbourne, Victoria, 3000, Australia

## 

Varying morphology of the peroneal tubercle can be associated with peroneal tendon pathology including tendonosis, stenosing tenosynovitis, tendon attrition and tears. The authors present a case of peroneus brevis rupture associated with an enlarged peroneal tubercle in a 59yo male. The clinical testing, medical imaging, diagnosis, surgical reconstruction and rehabilitation is discussed. The literature review results are also reported.

A literature review was conducted across several scientific databases including Medline, CINAHL and PubMed using the search terms “peroneus brevis” AND “rupture” AND “peroneal tubercle” OR “peroneal trochlea”. Further information was sought with citation tracking and reference checking and reviewing unpublished data.

No report of isolated peroneus brevis rupture associated with a hypertrophic peroneal tubercle was found in the literature. However, there were several reports of peroneal tendinopathy associated with this pathology. Failing conservative care, good to excellent results were reported with surgical resection and tendon repair. In our case resection of the tubercle and peroneus brevisrepair with a split thickness peroneus longus graft was performed. At 3 years post-op the patient was asymptomatic, had returned to previous level of activity and was satisfied with the result.

The hypertrophic peroneal tubercle can cause significant lateral foot pain. It is important for clinicians to keep this pathologic process in the list of differential diagnoses. X-rays (particularly the axial calcaneal view), ultrasound, CT and/or MRI can identify the extent of the pathology. If conservative measures fail, generous resection and repair of any peroneal tendinopathy can result in return to normal function.

